# First use of artificial canopy bridge by the world’s most critically endangered primate the Hainan gibbon *Nomascus hainanus*

**DOI:** 10.1038/s41598-020-72641-z

**Published:** 2020-10-15

**Authors:** Bosco Pui Lok Chan, Yik Fui Philip Lo, Xiao-Jiang Hong, Chi Fung Mak, Ziyu Ma

**Affiliations:** 1 Kadoorie Conservation China Department, Kadoorie Farm & Botanic Garden, Lam Kam Road, Tai Po, N.T. Hong Kong; 2 Hainan Bawangling National Nature Reserve, Changjiang County, Hainan, China; 3 c/o: Kadoorie Conservation China Department, Kadoorie Farm & Botanic Garden, Lam Kam Road, Tai Po, N.T. Hong Kong

**Keywords:** Ecology, Zoology

## Abstract

All gibbon species (Primates: Hylobatidae) are facing high extinction risk due to habitat loss and hunting. The Hainan gibbon *Nomascus hainanus* is the world’s most critically endangered primate, and one of the priority conservation actions identified is to establish artificial canopy corridors to reconnect fragmented forests. The effectiveness of artificial canopy bridge as a conservation tool for wild gibbons has not been widely tested, and the results are rarely published. We constructed the first canopy bridge for Hainan gibbon in 2015 to facilitate passage at a natural landslide; mountaineering-grade ropes were tied to sturdy trees with the help of professional tree climbers and a camera trap was installed to monitor wildlife usage. Hainan gibbon started using the rope bridge after 176 days, and usage frequency increased with time. All members in the gibbon group crossed the 15.8 m rope bridge except adult male. Climbing was the predominant locomotor mode followed by brachiation. This study highlights the use and value of rope bridges to connect forest gaps for wild gibbons living in fragmented forests. While restoring natural forest corridors should be a priority conservation intervention, artificial canopy bridges may be a useful short-term solution.

## Introduction

Habitat fragmentation is one of the major causes of biodiversity loss^1–5^. The impact is particularly detrimental to arboreal wildlife, as forest gaps affect dispersal, foraging and energy efficiency, and breeding opportunity^6–9^, increase vulnerability to predation, diseases and parasites, accidental fatalities such as roadkill and electrocution^10–16^, resulting in an increased risk of extirpation in isolated populations^17–19^. To mitigate fragmentation impacts, artificial aerial wildlife crossing structures, commonly known as artificial canopy bridges, are being increasingly built worldwide to re-establish canopy connectivity^20^, which is more prevalent in South America and Australia^12,20–25^.

The great expanse of Asian tall forests supports a diverse assemblage of arboreal wildlife^26,27^, which are subjected to multiple threats including some of the highest forest fragmentation rates in the world^5^. Asia is the second richest continent for primate diversity after the Neotropics^26^, but the use of artificial canopy bridges as a conservation intervention is limited, and the effectiveness of these attempts are rarely examined and documented^16^. Artificial canopy bridges have been constructed for orangutan (*Pongo pygmaeus*)^28,29^, western hoolock gibbon (*Hoolock hoolock*)^7,30^, Javan slow loris (*Nycticebus javanicus*)^16^and dusky langur (*Trachypithecus obscurus*)^31^, and in forests bisected by roads in South Asia^32,33^.

Gibbons (Primates: Hylobatidae) are a group of highly specialised Asian apes which live in social groups and form well-established territories^34^. Canopy connectivity is critical for gibbons as they are strictly arboreal travel mainly through continuous tree canopy, forest fragmentation thus presents a major conservation challenge for gibbons^7,14,34–36^. The construction of artificial canopy bridge for gibbon conservation has not been widely experimented and its success rate rarely documented. Only two successful cases have been reported: lar gibbon in Thailand used rope bridges to cross a road^37^, and Western hoolock gibbon in India used bamboo and rope bridges to travel between isolated forest patches^7,30^. The HUTAN Kinabatangan Orang-utan Conservation Programme (KOCP) in Sabah constructed various types of artificial canopy bridges for primates in fragmented forest, which have been used by the Müller’s gibbon (*Hylobates muelleri*) (Boonratana, R., pers. comm. 2020). The current IUCN Red List of Threatened Species categorized all 20 gibbon species as threatened with extinction, and the crested gibbons (genus *Nomascus*) of southern China and Indochina are particularly imperiled, with five of the seven assessed species listed as Critically Endangered, and the remaining two Endangered.

The Hainan gibbon (*Nomascus hainanus*) is endemic to China’s Hainan Island. It is the rarest primate species on Earth with around 30 individuals remaining for the entire species, all living in Hainan Bawangling National Nature Reserve (hereafter BWL)^38–40^. With its precarious status many conservation recommendations have been suggested by concerned scientists, one of the priorities identified is to build artificial canopy crossings to connect the gibbons’ fragmented habitat^41^. Since 2005, we have been monitoring the Hainan gibbon population in a long-term conservation programme^40^.

**Figure 1:**
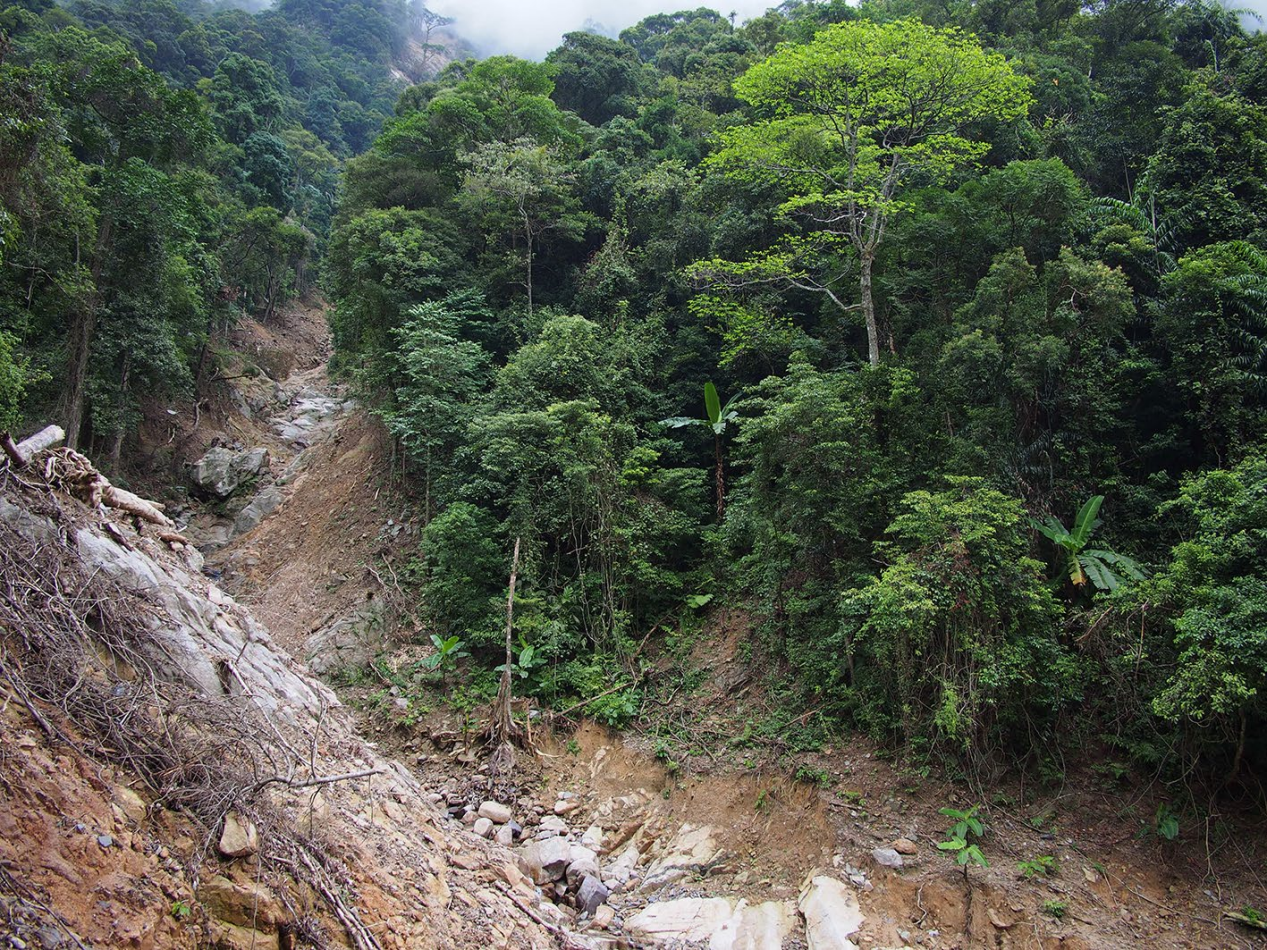
One of numerous landslides in the gibbon forest of Bawangling three months after Super Typhoon Rammasun, taken on 24 October 2014. Photograph taken by L.G.

In July 2014, Super Typhoon Rammasun, the strongest typhoon making landfall in Mainland China since 1949, hit Hainan Island and BWL received the highest rainfall in modern history (> 500 mm/24 h)^42^. Landslides scarred the forest-clad slopes throughout BWL, creating forest gaps up to 30 m wide (Fig. 1).

**Figure 2:**
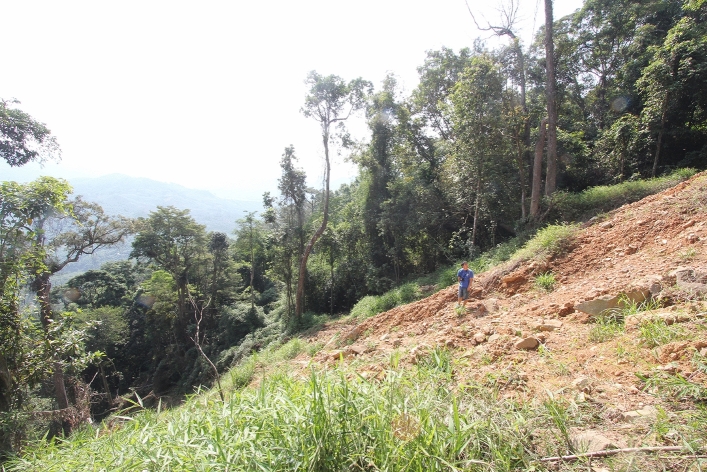
Forest gap of the canopy bridge site 10 months after Typhoon Rammasun, taken on 4 May 2015. Photograph taken by A.L.

One of the main ‘arboreal highways’^43^ of our study gibbon group, Group C, was damaged by a landslide, creating an open gully measuring 15 m wide on average (806–895 m asl, midpoint at 19° 05′ 47.28″ N, 109° 14′ 43.36″ E) (Fig. 2). The damaged arboreal highway is situated in the core home range of Group C, and was used on average every 2 days before the typhoon damage (KFBG unpubl. data). Subsequent to the typhoon, gibbons continued to use the broken ‘arboreal highway’ by leaping across the landslide with difficulty using fronds and leaves of *Arenga pinnata* along the open gully, or an alternative and more hazardous pathway by leaping over two widely-spaced surviving trees (*Elaeocarpus dubius* and *Castanopsis hystrix*, respectively) followed by a drop of 10 m to the sub-canopy (Supplementary Video S1 online). We observed gibbons showing signs of hesitation when attempting to cross the forest gap, especially the adult females and small juveniles. To avoid accidental injuries or deaths, we constructed a two-pronged canopy rope bridge across the damaged arboreal highway on 06 December 2015. Here, we contribute to understanding the value of canopy bridge as a conservation intervention for gibbons living in fragmented forests.

## Results

The gibbons were first photographed crossing the rope bridge 176 days after the bridge was placed. Data recording of the camera trap was intermittent due to camera malfunction and adverse weather preventing site access, and data were collected between 06 December 2015–2019 October 2016, 18 August–27 November 2018 and 06 January–25 February 2019. Throughout the study period of 470 days, we logged 208 photographs and 53 video footages of gibbons using the rope bridge (detailed information of all 52 crossing events can be found in Supplementary Table S1 online). During the 2015–2016 data collecting period, the camera trap angle failed to capture the crossing sequence, therefore only 48 out of the 52 crossing events, recorded during 2018 and 2019, could be used for analysis of locomotor behaviour. In the 2018–2019 study period, the gibbon group consisted of one breeding male, two breeding females, three large juveniles, two small juveniles and one infant. For analysis of locomotor behaviour, we did not consider the dependent infant attached to the mothers.

### Age–sex difference in locomotor mode of gibbon crossings

**Figure 3:**
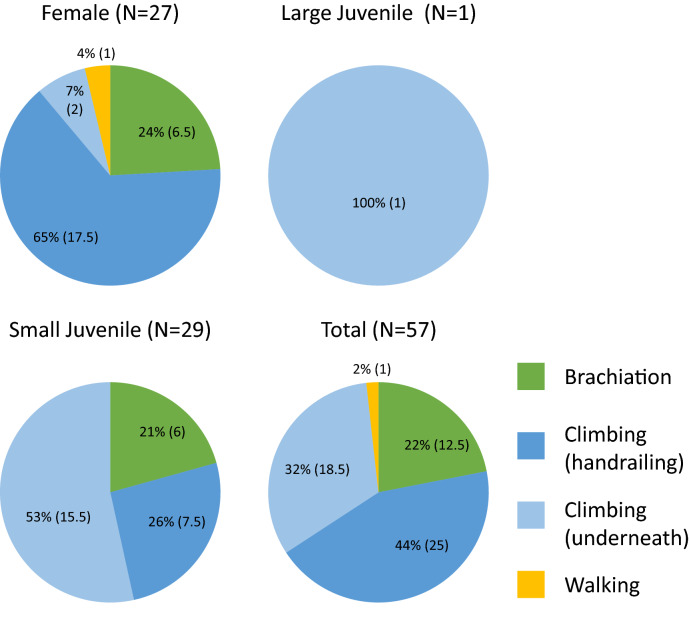
Age–sex difference in dominant form of locomotor mode on canopy bridge by the Hainan gibbon group. N is total number of individual crossing recorded by camera trap. Bridging was only used for entering and leaving the canopy bridge, and is excluded for analysis.

**Figure 4:**
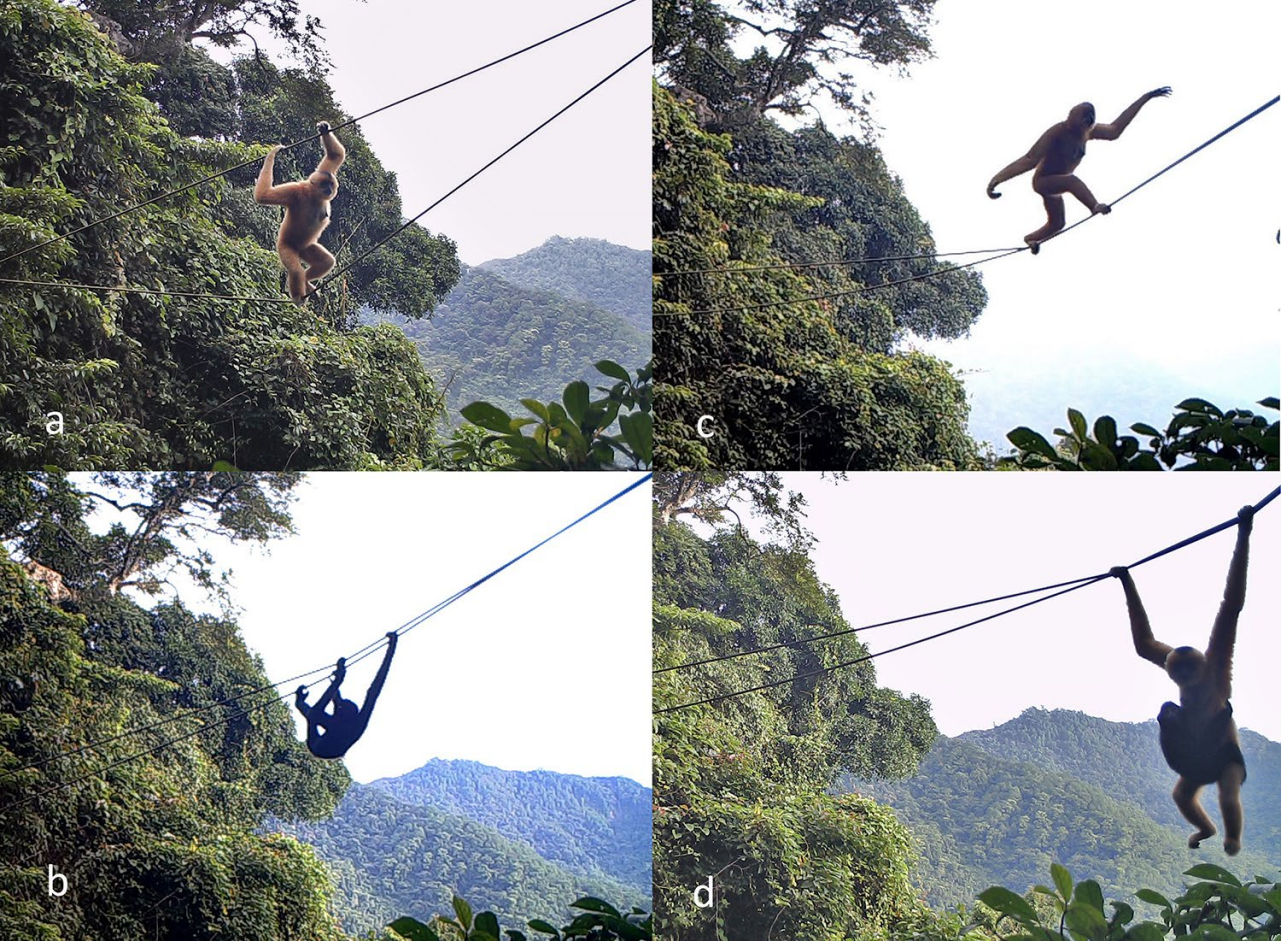
Locomotor modes of Hainan gibbons using a canopy bridge. (**a**) climbing (handrailing); (**b**) climbing (underneath); (**c**) walking; (**d**) brachiation. Camera trap photographs taken by Kadoorie Farm and Botanic Garden.

Gibbons used all five locomotor modes for crossing the artificial crossing, but bridging was only employed when mounting and descending the rope bridges via connecting trees and *Arenga pinnata*, and therefore was not counted in the locomotor behaviour analysis. Climbing was the most common locomotor mode (76% of all modes employed), followed by brachiation and rarely walking. Two types of climbing were used: ‘Handrailing’ is walking on one rope with hands holding the second rope as handrails, or climbing underneath the ropes legs first with all limbs. Most climbing involved handrailing (57% of all climbing recorded). Walking was rare and only successfully employed by adult females, with many failed attempts by juveniles. Small juveniles frequently employed a combination of climbing and brachiation, with climbing overwhelmingly used (79% of all data); underneath climbing was the most used locomotion (Fig. 3). All gibbon group members were recorded using the canopy bridge except the adult male, but large juveniles rarely used the canopy bridges and was recorded only once crossing the forest gap by rope. Instead the breeding male and large juveniles were repeatedly observed leaping across the forest gap by our monitoring team. See Fig. 4a–d for examples of different locomotor modes employed by Hainan gibbon on the canopy bridge, and Supplementary Video S2 online provides a video footage of different age–sex of Group C crossing the forest gap, using the canopy bridges as well as leaping.

### Frequency of gibbon crossings

For the three data collecting periods, the usage frequency increased as the gibbon group become habituated to the canopy bridge (Table 1).

**Table 1:** Usage frequency of artificial canopy bridge by the Hainan gibbon group during the three study periods in 2015–2019.

Recording period	No. of day	Number of crossing event	Frequency (event per day)
06 Dec 2015–19 Oct 2016	318	4	0.0126
18 Aug 2018–27 Nov 2018	102	27	0.2647
06 Jan 2019 –25 Feb 2019	50	21	0.4200

### Crossing sequence in a gibbon group

From our limited dataset, there was no well-defined age-sex selection in initiation of gap crossing. Half of the events were initiated by adult females, while the others were led by juveniles (see Supplementary Table S1 online).

### Usage by other mammals

Our camera traps also recorded the use of rope bridge by Pallas’s squirrel (*Callosciurus erythraeus*), a small flying squirrel (*Hylopetes* sp.) and a small rodent (Subfamily Murinae).

## Discussion

Forest fragmentation is one of the biggest threats to primate conservation^44^, and is becoming a primary concern in the study of primate conservation biology^45^. This study demonstrated the world’s rarest primate uses a simple artificial canopy bridge, and showed that artificial canopy bridge is a valuable conservation tool to reconnect fragmented gibbon habitats. The 15.8 m canopy bridge monitored was used for crossings in both directions, and both females and juveniles could initiate crossing.

A locomotor behaviour study of the cao-vit gibbon (*Nomascus nasutus*), the closest relative of Hainan gibbon, reported that adult females appear to employ safer locomotor modes. In comparison adult males choose riskier modes such as leaping^46^, which is in concordance with our observations as the adult male Hainan gibbon never, and near full-grown large juveniles rarely, used the canopy bridge over the study period. It is probable adults and near full-grown gibbons are more muscular and skilful to make big ricochetal brachiation or leap to overcome the forest gap. Simultaneously the forest gap presents too much an obstacle for the weaker and less skilful immatures. Adult females have similar physique to adult males, but it is likely leaping is too risky for pregnant and infant-carrying females with the extra weight. An alternative explanation is that innate maternal care behaviour resulted in the females always travel with their younger offspring using the rope bridges.

In areas where the target species are habituated to human presence, habituation to artificial crossings could be rapid: the western hoolock gibbon living in a village environs used a bamboo bridge in 15 days^7^, Javan slow loris in an agroforestry landscape used single-lined bridges after 20 days^16^, the western ringtail possum (*Pseudocheirus occidentalis*) around a major road with daily traffic volume up to 15,000 cars used the artificial bridge in 36 days^25^. It took the Hainan Gibbon 176 days to start using the canopy bridge, with a gradual increase in bridge use frequency from 2016 to 2019.

Different designs of artificial crossings for canopy connectivity have been constructed for wildlife: horizontal ‘ship’ ladder and pole bridge in Latin American^24^, box-tunnel bridge, rope ladder and netted flat bridge in Australia^22,25^, and simple rope and bamboo bridges in Asia^7,37^. It has been reported that target species could be selective on bridge type^16,47^, and bridges designed for gibbons are not always used, as canopy bridges erected for eastern hoolock gibbon (*Hoolock leuconedys*) in India for a translocation operation were never used^36^ , and the isolated population of western hoolock gibbons in northeast India never cross a metal canopy bridge until a natural tree canopy bridge was established after 13 years^48^. Rope bridges are inexpensive, material readily available, easy to transport in remote hilly regions, technically simple to make and install, and have been used by three of the four extant gibbon genera (*Nomascus*, *Hoolock* and *Hylobates*). We suggest it may be the most cost-effective artificial canopy bridge type for Hylobatidae, and should be constructed and tested in areas where gibbon habitat is fragmented.

Brachiation is the predominant locomotion mode during travel by gibbons^49^. It is possible the rope diameter for building our canopy bridge is not the most desirable for Hainan gibbon locomotion as climbing instead of brachiation was the most employed mode in our study. Gibbon locomotion studies in Malaysia show that Siamang *Symphalangus syndactylus*, Lar Gibbon *Hylobates lar* and Agile Gibbon *H. agilis* tend to brachiate on larger support and climb on smaller ones^49^, and support between 20–100 mm diameter was most frequently used (> 60%) for a similar study on cao-vit gibbon^46^. Our flexible canopy bridge was constructed using 13 mm diameter ropes and may not be providing the most desirable support for brachiation in Hainan gibbon. However, one must consider the logistic and financial implications, as well as the sustenance of supporting trees for choosing thicker ropes or more rigid materials for building canopy bridges.

We considered the canopy bridge as a temporary measure in the conservation management of Hainan gibbon, and launched a reforestation project at the landslide after completion of the canopy bridge construction as a long-term solution to restore forest connectivity. Twenty-five large saplings (> 150 cm tall) of a fast-growing native tree, *Bishchofia javanicus*, a favourite gibbon food tree with large crown, were planted underneath the canopy bridge. Two years after the landslide, dense growth of pioneering trees, e.g. *Ficus esquiroliana, Broussonetia papyrifera*, *Trema orientalis* and *Macaranga denticulate* also regenerated underneath the rope bridges, and together with the planted saplings, they have been providing an alternative crossing route for the gibbons since November 2018. The lifetime of climbing ropes varies depending on use frequency and manufacture specifications, but a general replacement guideline for human usage is 3–5 years (Cho Wing Chung, pers. comm., 2020). Although gibbons weigh a lot less than human and the ropes should last longer than the safety standard for human usage, it was our plan to replace the ropes in 2020. However, the COVID-19 pandemic precluded traveling thus replacement. The condition of the ropes has been visually monitored regularly by our gibbon monitoring team for signs of external wear and tear. Although we cannot assess fatigue in the ropes, the dense growth of regenerated vegetation underneath the rope bridge is believed to provide a safe landing platform for the gibbons should an accidental fall happened. We recommend building canopy bridges at the early stage of a forest gap restoration project to provide temporary connectivity, but reforestation with fast-growing native trees should also be simultaneously carried out as a long-term solution where it is socioeconomically feasible.

## Methods

### Study area

**Figure 5:**
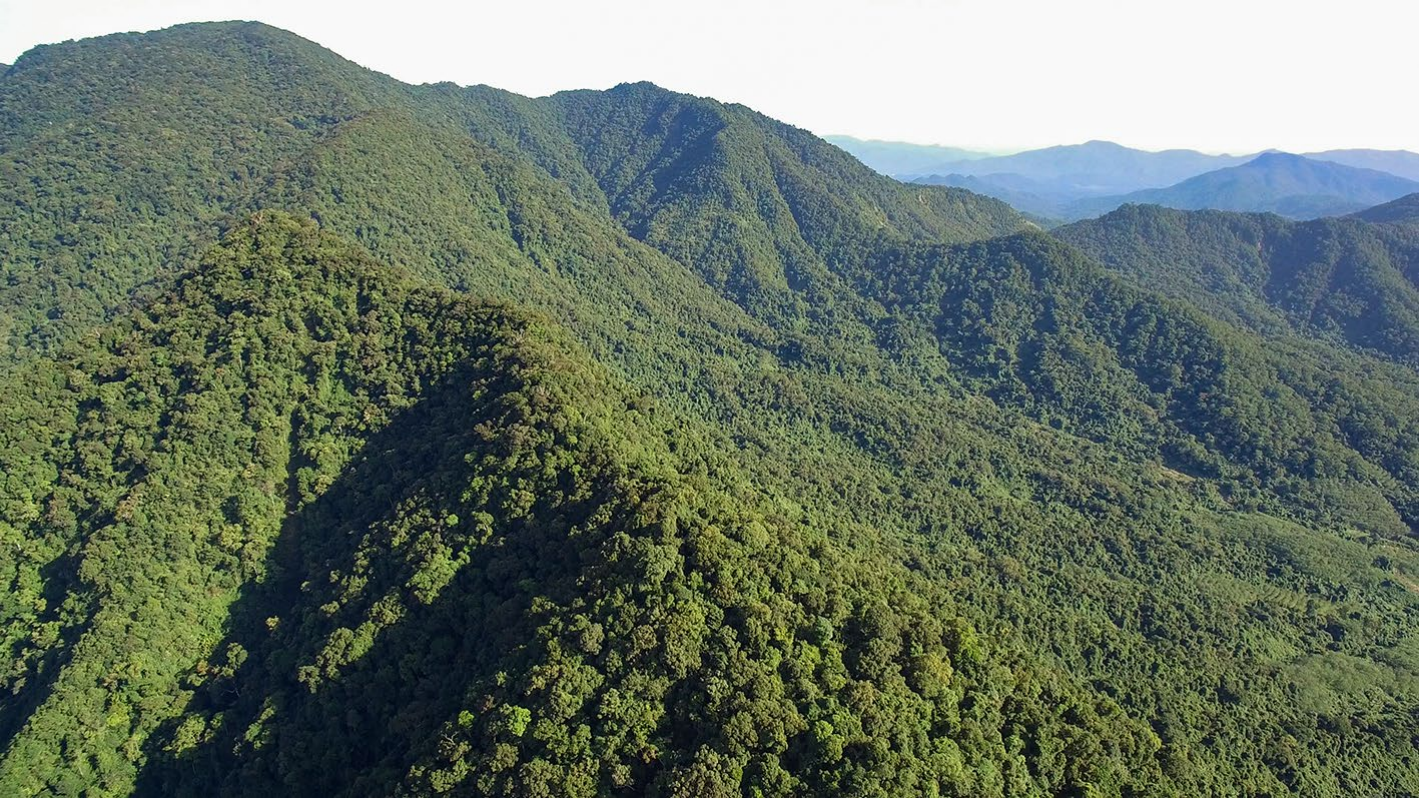
Landscape of the study area (Xiaofutouling is in the foreground), taken on 21 June 2018. Photograph taken by Z.M.

Xiaofutouling is a peak (1030 m asl) on the eastern border of BWL, and the zonal vegetation is seasonal tropical rainforest. The forest of Xiaofutouling was selectively logged in the 1970s and subjected to intense human use due to its proximity to villages, but enhanced patrolling effort in the last nine years has substantially curbed human disturbance. Despite these sustained disturbances, the mountain is still covered by contiguous old-growth forest with a canopy height of 25 m above ca. 700 m asl (Fig. 5). Common tree species include *Castanopsis* spp., *Elaeocarpus* spp., *Endospermum chinense*, *Ficus* spp., *Liquidambar formosana*, *Lithocarpus* spp., *Nephelium topengii* and *Pinus latteri*, some of which are important gibbon food tree species.

### The study gibbon group

The study group, Group C, was formed in 2011 and started breeding in 2013, gradually becomes the largest gibbon group in BWL. At the onset of the study, Group C consisted of eight gibbons including one breeding male, two breeding females, one large juvenile and four small juveniles. With three births and two individuals dispersed by late 2018, Group C had nine gibbons (one breeding male, two breeding females, three large juveniles, two small juveniles and one infant) at the end of the study. Survivorship of immature gibbons had been 100% according to our long-term monitoring data. The group has been habituated by a gibbon monitoring team we trained since 2011, allowing detailed observation during daily tracking.

### The canopy bridges

**Figure 6:**
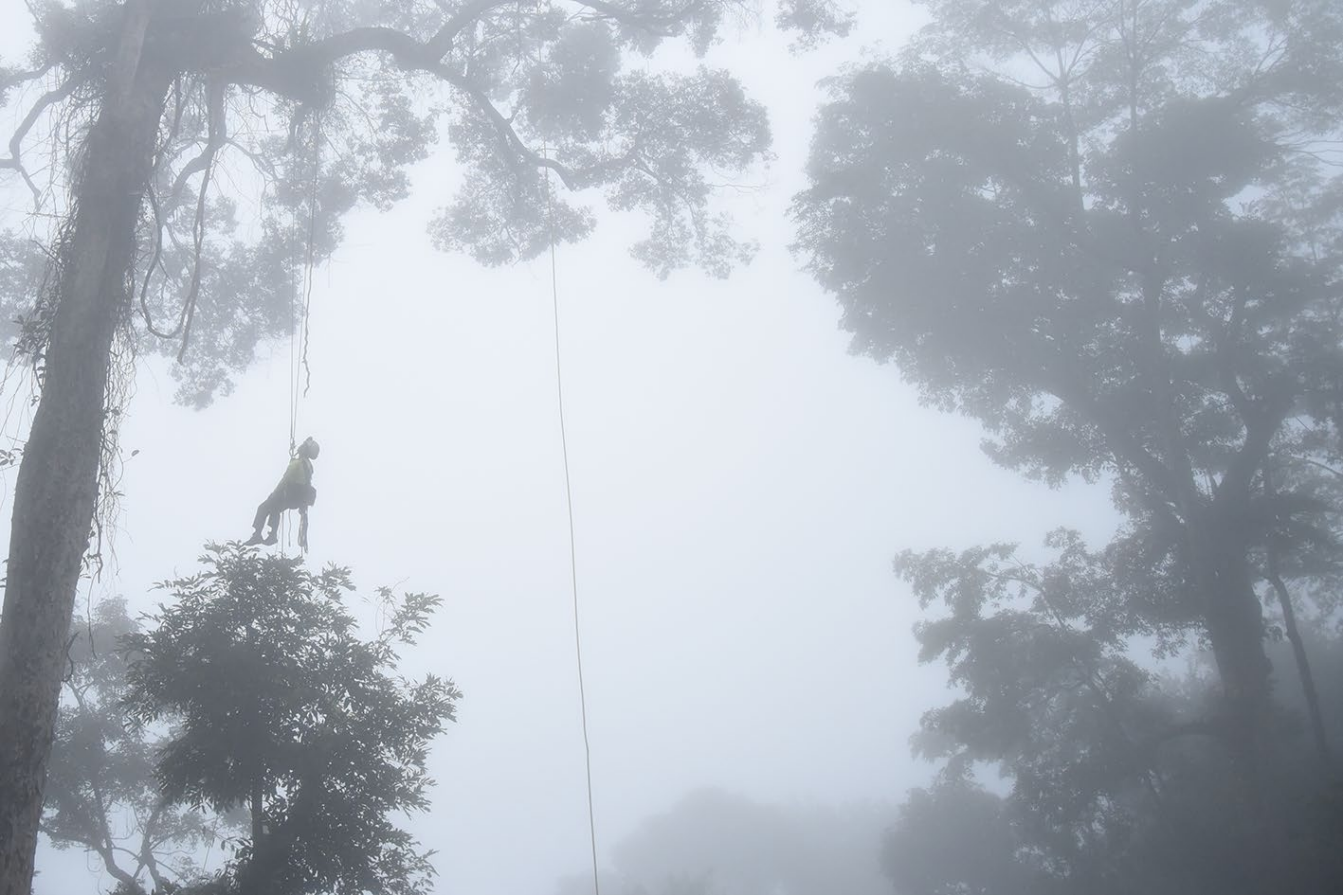
Installation of the canopy bridge by professional tree climbers, taken on 06 December 2015. Photograph taken by C.F.M.

**Figure 7:**
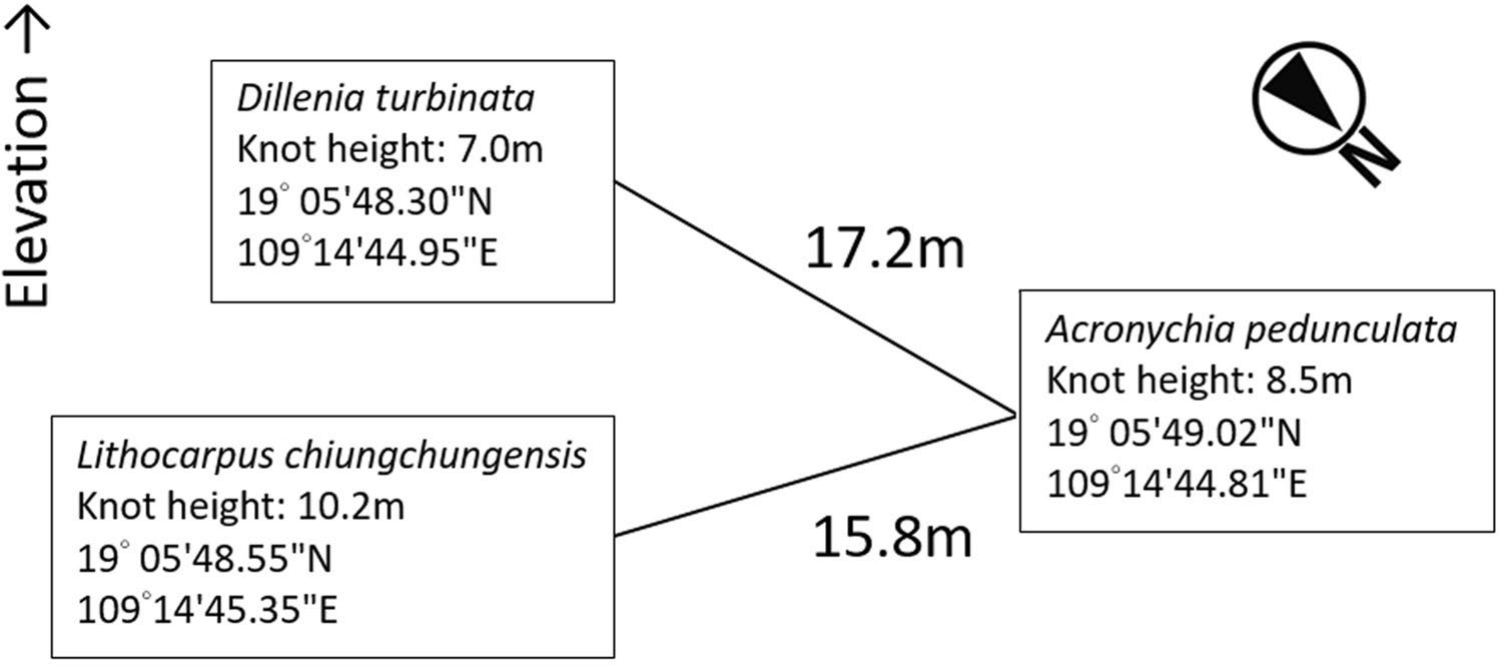
A schematic diagram of the canopy bridge for Hainan gibbon showing GPS coordinates, rope length, knot height and species of supporting trees.

With consideration to the unique brachiation locomotion of gibbons, we designed a simple rope bridge, which is technically and economically low-cost, and minimize the risk of entanglement during brachiation. Since canopy bridge construction at the study site involved much logistical challenges, including 3 h of uphill hiking, camping and technical tree climbing on steep terrain, we designed a two-pronged, double-roped canopy bridge network, making sure a back-up rope is always available at the damaged arboreal highway. On 06 December 2015, three professional tree climbers constructed the canopy bridge at 824 m asl (19 °05′ 48.3″ N, 109° 14′ 44.9″ E) (Fig. 6). Each bridge consisted of two mountaineering-grade 13 mm diameter ropes tied at the same knot on sturdy trees at the natural gibbon crossing. One bridge measures 15.8 m in length and the other 17.2 m (Fig. 7). Distances and tree heights were measured by a Nikon Forest Pro laser rangefinder. The total cost of constructing the canopy bridge was ca. USD $5000, including raw materials, service fee of professional tree climbers and logistical cost of reaching the remote site.

### Crossing monitoring

In order to evaluate the effectiveness of the artificial canopy bridge for Hainan gibbon, we monitored the rope bridge by installing a camera trap from 06 December 2015 (Loreda L710 [December 2015 to August 2018] and ScoutGuard SG-990V [August 2018 to February 2019]). Once triggered, the camera trap recorded three still images followed by a 10-s video. We identified separate crossing events by all crossings recorded within a 5-min period based on the time and date of photographs/videos taken by the camera trap; thus it could be a single gibbon crossing or multiple individuals crossing in quick succession. As our long-term ground monitoring data show that members within the same gibbon group always travel together, our study provided data on the age–sex difference in locomotor behaviour of Hainan gibbon using artificial canopy bridge.

To avoid any potential problem induced by temporal autocorrelation, we used camera trap video footages to study the dominant form of locomotion of each individual in crossing the rope bridge to analyse the age-sex difference in locomotor mode of Hainan gibbon. We categorized five different locomotion modes in the present study following Fan et al.^46^: (i) brachiation, which is arm-over-arm suspensory progression, which can be rapid and ricochetal; (ii) climbing, which is any continuous progression involving three or more limbs; (iii) bridging, which is a special form of climbing in which the animal crosses a small discontinuity in the canopy; (iv) walking, bipedal locomotion along a support with arms held out for balance; and (v) leaping, where the animals launches themselves by pulling with the arms, and usually lands on all four limbs, and dropping was included in leaping. We also recorded sequence of crossing by different age and sex in the gibbon group; we followed the same publication for age-sex categorizations, large juveniles were distinguished from small juveniles based on body size, being approaching the size of but smaller than full-grown adults.

Informed consents have been obtained from the persons in Figs. 2 and 6 to publish the images in an online open-access publication.

## Supplementary information

Supplementary Table S1.

Supplementary Legends.

Supplementary Video S1.

Supplementary Video S2.
