# Supplementary material for: First use of artificial canopy bridge by the world’s most critically endangered primate the Hainan gibbon Nomascus hainanus
Source: Sci Rep. 2020 Oct 15;10:15176. doi: 10.1038/s41598-020-72641-z (PMC7567071; doi:10.1038/s41598-020-72641-z)
Supplement: Supplementary file 1 — Supplementary Table S1. [file 41598_2020_72641_MOESM1_ESM.pdf]

First use of artificial canopy bridge by the world's most critically endangered primate the Hainan gibbon *Nomascus hainanus*

Authors: Bosco Pui Lok CHAN, Yik Fui Philip LO, Xiao-Jiang HONG, Chi Fung MAK, Ziyu MA

Supplementary Table S1 online Date, travel direction and age–sex crossing sequence of all crossing events by the Hainan gibbon group during the study period in 2015-2019.

Crossing event	Year	Month	Day	Travel direction	Age–sex crossing sequence
**1**	2016	5	2	Uncertain	Uncertain
**2**	2016	7	30	Leaving	Uncertain
**3**	2016	8	17	Uncertain	Uncertain
**4**	2016	10	5	Uncertain	Uncertain
**5**	2018	8	25	Coming	large juvenile > female
**6**	2018	8	31	Leaving	female > small juvenile > large juvenile
**7**	2018	9	3	Coming	small juvenile > female
**8**	2018	9	8	Leaving	small juvenile 1 > female
**9**	2018	9	12	Leaving	small juvenile > small juvenile > female
**10**	2018	9	13	Coming	female > small juvenile
**11**	2018	9	13	Coming	female > small juvenile 1 > small juvenile 2
**12**	2018	9	13	Leaving	female > small juvenile 1 > small juvenile 2
**13**	2018	9	20	Leaving	female > small juvenile
**14**	2018	9	20	Coming	female
**15**	2018	9	22	Leaving	small juvenile > small juvenile > female
**16**	2018	9	25	Coming	small juvenile > female
**17**	2018	10	5	Leaving	female > small juvenile
**18**	2018	10	7	Leaving	small juvenile > female
**19**	2018	10	11	Leaving	female > small juvenile
**20**	2018	10	14	Leaving	small juvenile > female > small juvenile
**21**	2018	10	20	Coming	small juvenile > female
**22**	2018	10	25	Coming	small juvenile > female
**23**	2018	10	28	Coming	female
**24**	2018	10	31	Leaving	small juvenile > female
**25**	2018	11	2	Coming	female
**26**	2018	11	4	Leaving	female > small juvenile
**27**	2018	11	7	Leaving	female > small juvenile 1 > small juvenile 2
**28**	2018	11	11	Coming	small juvenile 1 > small juvenile 2 > female
**29**	2018	11	14	Coming	small juvenile 1 > female > small juvenile 2
**30**	2018	11	22	Leaving	small juvenile 1 > small juvenile 2 > female

**31**	2018	11	27	Leaving	female > small juvenile
**32**	2019	1	8	Leaving	female
**33**	2019	1	8	Leaving	small juvenile
**34**	2019	1	10	Coming	female > small juvenile 1 > small juvenile 2
**35**	2019	1	14	Leaving	small juvenile 1 > female > small juvenile 2
**36**	2019	1	14	Coming	small juvenile 1 > female > small juvenile 2
**37**	2019	1	15	Leaving	small juvenile > female
**38**	2019	1	16	Coming	small juvenile 1 > small juvenile 2 > female
**39**	2019	1	18	Leaving	female
**40**	2019	1	21	Leaving	small juvenile > female
**41**	2019	1	21	Coming	female
**42**	2019	1	25	Coming	female > small juvenile 1 > small juvenile 2
**43**	2019	1	27	Leaving	female > small juvenile 1 > small juvenile 2
**44**	2019	1	30	Leaving	small juvenile 1 > feamale > small juvenile 2
**45**	2019	1	30	Leaving	female
**46**	2019	2	3	Leaving	female > small juvenile
**47**	2019	2	7	Coming	small juvenile
**48**	2019	2	8	Coming	female
**49**	2019	2	12	Leaving	female > small juvenile 1 > small juvenile 2
**50**	2019	2	14	Leaving	female > small juvenile
**51**	2019	2	19	Coming	small juvenile 1 > female > small juvenile 2
**52**	2019	2	25	Coming	small juvenile 1 > small juvenile 2
