# Supplementary material for: First use of artificial canopy bridge by the world’s most critically endangered primate the Hainan gibbon Nomascus hainanus
Source: Sci Rep. 2020 Oct 15;10:15176. doi: 10.1038/s41598-020-72641-z (PMC7567071; doi:10.1038/s41598-020-72641-z)
Supplement: Supplementary file 2 — Supplementary Legends. [file 41598_2020_72641_MOESM2_ESM.docx]

**Supplementary Video S1 online** Video footage of Hainan gibbons crossing the forest gap created by a natural landslide, taken on 20 May 2015 by Canon PowerShot SX60HS.

**Supplementary Video S2 online** Video footage of Hainan gibbons crossing the artificial canopy bridge, taken on 22 April 2020 by Canon PowerShot SX60HS.
